# Case report: Treatment of left-sided valve endocarditis using the Transapical AngioVac System and cerebral embolism protection device: A case series

**DOI:** 10.3389/fcvm.2023.1121488

**Published:** 2023-03-30

**Authors:** Alessandro Fiocco, Andrea Colli, Laura Besola

**Affiliations:** Department of Surgical, Cardiac Surgery Unit, Medical and Molecular Pathology and Critical Care, University of Pisa, Pisa, Italy

**Keywords:** valve endocarditis, AngioVac System, TriGuard embolic protection device, minimally invasive, transapical access, prosthesis endocarditis

## Abstract

The AngioVac System (AngioDynamics, Latham, NY) was developed for the treatment of right-sided heart and intravenous masses. Lately, it has been employed to deal with left-sided heart masses, in particular, native valve endocarditis (NVE) and valve prostheses endocarditis (VPE) in high-risk patients. Left-sided heart endocarditis has a high morbidity, and it also has a high mortality when open heart surgery is performed. Recently, patients presenting with left NVE and VPE have been treated with the off-label use of the AngioVac System even if the solution presents a considerable cerebral embolization risk issue due to the risk of fragmentation rather than a complete en-bloc aspiration of the masses. A percutaneous cerebral embolism protection system is currently used in TAVI procedures, especially when the native valve presents extensive calcifications and consequent significant embolic risks. We hereby present a clinical case series of a combined utilization of the AngioVac System and cerebral embolism protection system Triguard (Keystone Heart Ltd., Herzliya, Israel) to treat left NVE and VPE in prohibitive-surgical-risk patients.

## Introduction

The incidence of mitral or aortic endocarditis either on the native valve or prosthesis has increased over the last decade (1). Surgery is used in the presence of acute heart failure following valve dysfunction, local tissue destruction, large vegetations, and persistent bacteremia despite optimal prolonged antibiotic therapy (2). When not treated, infective endocarditis has high morbidity and mortality particularly when it involves valve prosthesis (1). However, approximately 20% of patients are not referred for surgery mainly because of their high surgical risk (3). The AngioVac System has FDA approval and a CE mark for the sole treatment of soft masses and embolic material in the right heart. It consists of a suction cannula, an extra-corporeal circuit including a filter, and a reinfusion cannula. Recently, off-label use of this technology has been prescribed for left-sided masses removal (4–6) even with concerns about cerebral embolization. Cerebral embolization prevention systems (TriGuard, Keystone Heart Ltd., Herzliya, Israel; Sentinel, Boston Scientific) are occasionally used in heavy calcified native aortic valves during TAVI procedures. In this study, we describe two cases of left-sided endocarditis treated with transapical AngioVac vegetation aspiration coupled with the positioning of a cerebral embolic prevention device.

## Patient #1 native mitral valve endocarditis

**Figure 1:**
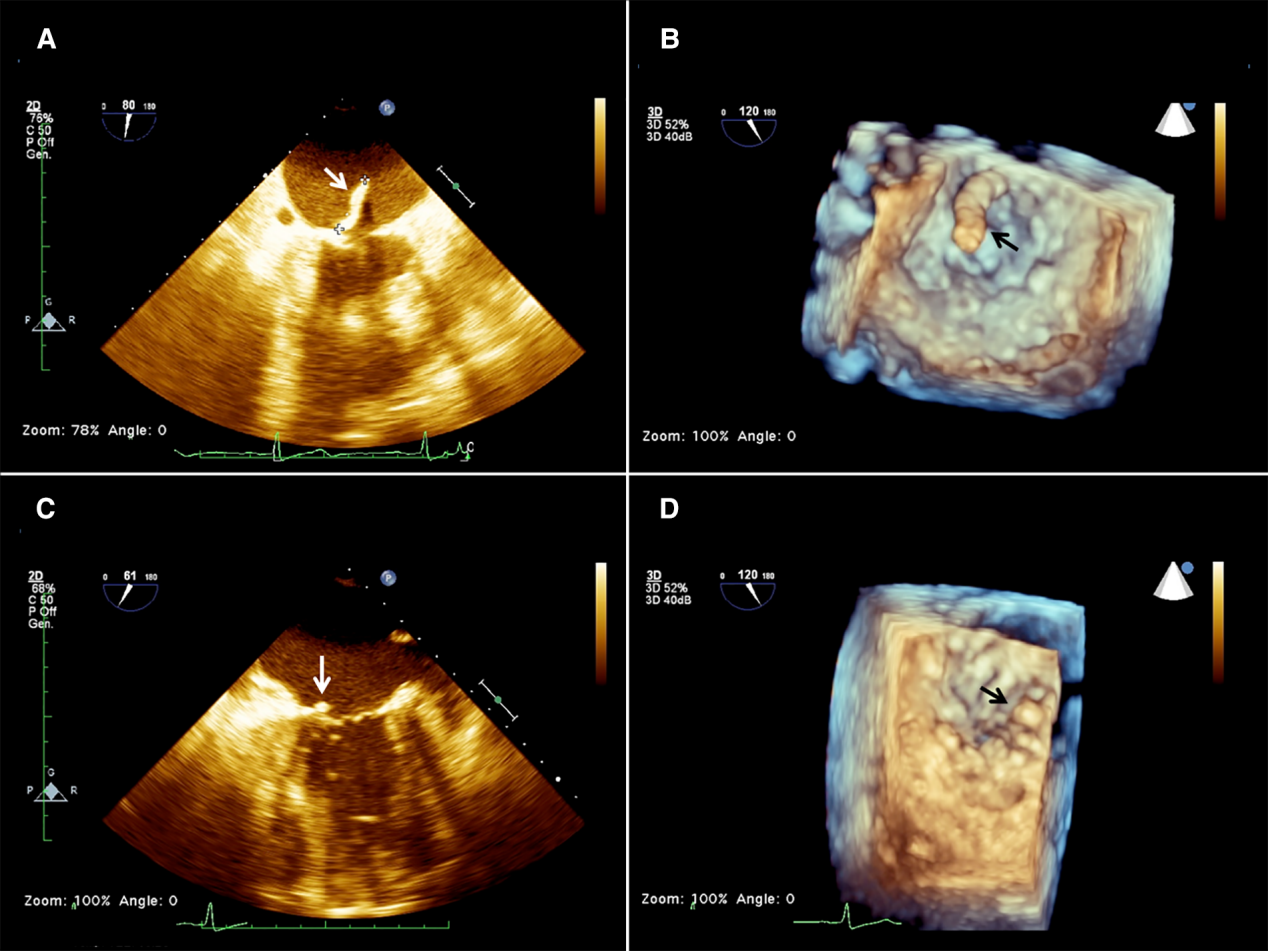
2d (**A**) and 3D (**B**) preoperative transesophageal echocardiography (TEE) showing the presence of a mass (arrow) attached to the base of the anterior mitral leaflet on the atrial aspect of the mitral valve, and 2D (**C**) and 3D (**D**) postoperative TEE showing a residual minimal stump (arrow).

A 57-year-old woman was brought to the emergency room after an incidental fall due to loss of balance. She had a history of insulin-dependent type 2 diabetes complicated with inferior limbs trophic ulcers. She was also on hemodialysis for chronic kidney disease and was being treated for peripheral vasculopathy with right leg arteries and renal arteries stenting. She had severe obesity and was newly diagnosed with a left kidney mass with surgical indications. Her symptoms included bilateral hip pain, exertional dyspnea, fatigue, and drowsiness. Blood tests did not show significant alterations except for anemia, a critical increase in the white cell count (21.80×*10^3^/µl), and inflammatory markers (CRP 19.63 mg/dl and 2.96 ng/ml procalcitonine). Creatinine was 7.47 mg/dl and urea was122 mg/dl. A transthoracic echocardiography (TTE) showed a large floating mass attached to the atrial surface of the posterior leaflet of the mitral valve close to the annular leaflet insertion. The mass freely prolapsed into the ventricular chamber during diastole, showing a mobile behavior and a small implant basis. Mild-to-moderate mitral regurgitation was associated with mass prolapse. These findings were later confirmed by transesophageal echocardiography (TEE), measuring a 20 × 8 mm neoformation (Figures 1A,B).

Considering her high surgical risk, the patient was accepted for mass aspiration using the AngioVac system. A cerebral embolic protection device TriGuard (Keystone Heart Ltd., Herzliya, Israel) was used before the procedure. The left subclavian artery was exposed and cannulated with a 16 Fr cannula (Biomedicus, Medtronic, Minneapolis, MN, USA) for blood reinfusion. Transapical access was obtained through a left anterolateral mini-thoracotomy at the 5th intercostal space. Two perpendicular pledgeted U-shaped purse strings were placed at the entry site. After full heparinization, to reach an ACT above 450 s, the ventricle was punctured, and an extra-stiff guidewire was inserted in the left ventricle (LV), carefully crossing the MV under real-time bi-plane TEE guidance. A 26 French GORE DrySeal (W.L. Gore & Associates, Newark, DE) was inserted on the guidewire into the left ventricle and was used to advance a 22 French 180 degrees AngioVac aspiration cannula. The circuit was then established by connecting the outflow to the apical suction cannula and the inflow to the subclavian arterial cannula. An oxygenator (Horizon, Eurosets, Medolla, Italy) was interposed in the circuit, distally to the filter and to the centrifugal pump. The AngioVac cannula was maintained below the MV plane, and the suction was initiated till the mass disappeared on the TEE image and only the stump was left (Figures 1C,D). The suction cannula and the sheath were withdrawn from the heart, reinfusion of the blood was completed, the subclavian arterial cannula was removed, protamine was administered, and purse strings were tied. The TriGuard device was finally retrieved from the right femoral artery which was closed using a percutaneous suture-mediated closure system (PercloseProGlide SMC System, Abbot Vascular, CA, USA). The patient remained hemodynamically stable during the whole procedure with minimal blood loss. At extubation time in the operating room (OR) her neurological status was intact and no bowel/limb ischemia was observed. No specimens were available for histologic examination. Since we could not ascertain the true nature of the mass, intravenous antibiotic therapy was carried on for 6 weeks as part of the endocarditis protocol. TTE was performed 1 week after the procedure, and mild mitral regurgitation (MR) and no regrowth of the mass were reported.

## Patient #2 mitral and aortic bioprosthesis endocarditis

**Figure 2:**
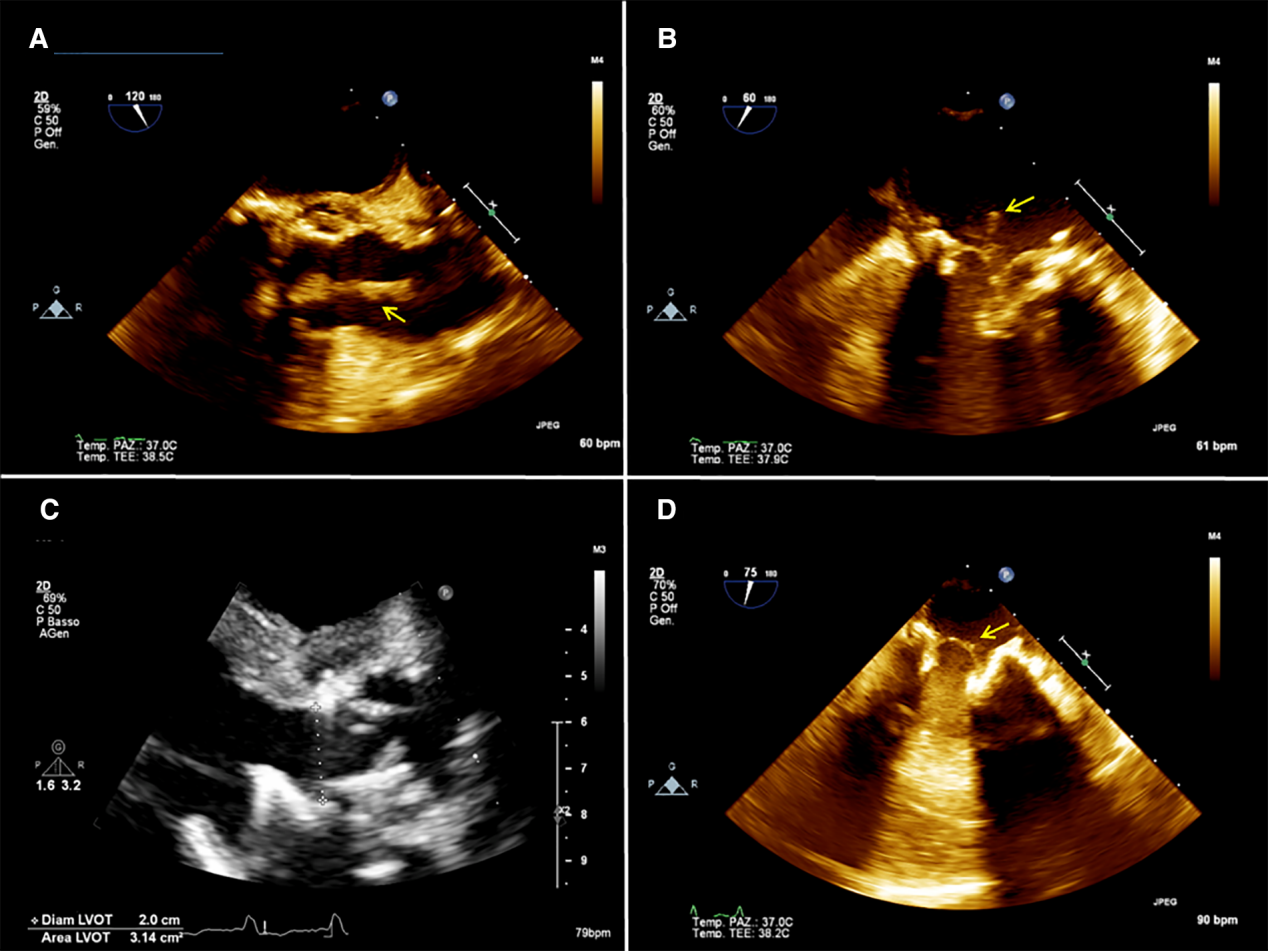
(**A**) 2D preoperative transesophageal echocardiography (TEE) showing the presence of a mass (arrow) attached to the aortic prosthesis and floating in the aortic root. (**B**) 2D preoperative TEE showing a mass (arrow) attached to the mitral prosthesis on the atrial side. (**C**) postoperative 2D TEE showing no residual mass on the aortic prosthesis. (**D**) postoperative 2D TEE showing a residual minimal stump (arrow) on the mitral prosthesis.

A 54-year-old man with a history of previous aortic valve replacement with a mechanical prosthesis in 2018 and aortic and mitral valve replacement with bioprosthesis, both following valve endocarditis and permanent pace-maker implantation and intravenous (IV) drug abuse was brought to the emergency department for severe asthenia. On physical examination, he was sarcopenic and his vitals were normal. His blood test revealed anemia and kidney dysfunction. Considering his cardiac medical history, he underwent a TTE and a TEE that showed moderate biventricular dysfunction and the presence of vegetation on the mitral and aortic prosthesis with no regurgitation or signs of valve dysfunction (Figures 2A,B). Blood cultures tested positive for Enterococcus Faecalis, and IV antibiotic therapy was started. A total body CT scan showed spleen embolization. Because of the prohibitive risk related to the patient's poor general conditions, the complexity of the potential surgical correction of the disease, and the ongoing IV drug use, the AngioVac system was used to perform aspiration on the patient. A TriGuard was inserted through the left common femoral artery at the beginning of the procedure, then the left subclavian artery was isolated and cannulated with a 16 Fr Biomedicus cannula. The heart apex was exposed and prepared as previously described; a 26 French GORE DrySeal was inserted over a guidewire, and under real-time 2D bi-plane and 3D TTE, the 22 Fr 180° AngioVac cannula was connected to the circuit (with the same setup used for patient 1) and advanced just below the aortic plane and suction was initiated until most of the vegetations disappeared. With real-time 2D bi-plane and 3D TEE guidance, we crossed the mitral prosthesis with the AngioVac cannula; the cannula was bent to 180° and suction was started until satisfactory aspiration of the mitral vegetations was achieved. The final TEE showed no significant residual mass, trivial intraprosthesis aortic, and mitral regurgitation (Figures 2C,D). The procedure was completed as described above. The TriGuard was removed in the standard fashion and small vegetation fragments were found and sent for a culture test. The patient was extubated in the OR, and he did not report any neurological impairment or bowel and limb ischemia. At 1 month, TTE showed partial detachment of the aortic prosthesis with a moderate paravalvular leak (PVL) and absence of new vegetation. Considering the patient's surgical risk and good hemodynamic conditions, we preferred conservative management. Six-month TTE showed no leak progression and no vegetation.

## Discussion

The AngioVac System has become a viable alternative to surgery to treat tricuspid valve/prosthesis and intravenous leads for permanent rhythm devices in patients with high surgical risk (7–9). Recent reports (10–12) showed its safety and effectiveness in different scenarios mostly involving the venous system and the right heart chambers, demonstrating its versatility as an option also to treat high-risk patients. More than having complete control of the infection, the first aim of the procedure is to debulk the vegetation size, lowering the embolic risk and the microbic burden with a positive impact on the effect of antibiotic therapy, thereby enhancing its effectiveness and controlling systemic involvement (9).

Treatment of aortic or mitral prosthesis endocarditis with the AngioVac System, though a transapical or transeptal approach, has been previously described with encouraging results (4–6). In this study, we report the first cases of the native mitral valve and combined mitral and aortic prosthesis endocarditis treatment using the AngioVac System in combination with a cerebral embolic protection device.

The presence of a floating mass attached to one of the left-sided valves of the heart is an urgent indication for cardiac surgery because of the high embolic risk (2). Conventional surgery entails the need for a cardiopulmonary bypass (CPB) and cardioplegic arrest which represent a great threat to frail patients like the reported cases. Therefore, a minimally invasive, beating-heart solution to remove the mass represents a valid alternative when there is no significant valve regurgitation or destruction.

Gerosa et al. (4) reported the use of the AngioVac System to treat an endocarditic mass located on the ventricular side of a mitral bioprosthesis through a transapical surgical approach. On the basis of these reports, we decided to extend the use of the AngioVac system to treat infective endocarditis involving the native mitral valve and both aortic and mitral prostheses. Both our patients were discharged with no in-hospital complications and no recurrence of endocarditis. The presence of a PVL at follow-up (FU) in Patient 2 was carefully evaluated since this condition is associated with worse outcomes; however, the decision for conservative management was driven by the patient's prohibitive surgical risk, which was the first reason we preferred to use the AngioVac procedure over conventional surgery. In cases like these, the PVL AngioVac procedure should be reserved for inoperable patients and close clinical FU is necessary. When treating the mitral valve, one drawback is an increased risk of MV subvalvular apparatus damage during LV navigation. Using a totally ventricular approach without crossing the valve under accurate real-time TEE guidance to enable optimal alignment with the MV orifice might lower this risk. Alternatively, a transeptal approach has been described (5, 6). However, after using the transeptal approach, the iatrogenic septal atrial defect might need to be closed with a closure device, but this occurrence is rare. Placement of material inside the heart in patients should, in our opinion, be avoided in patients with bacteremia; therefore, a careful evaluation of the hemodynamic impact of the ASD (significant shunt) must be done before proceeding with its closure. Transapical access, in expert hands, is a safe maneuver with minimal risk of access site complications and no significant impact on ventricular function (13), and the surgical technique to perform it is well established (14).

In the case of double involvement of mitral and aortic prostheses, a transapical approach allows for the corresponding treatment of both valves. In the presence of vegetation on the atrial side of a mitral prosthesis, it may be wise to prefer a 180° AngioVac cannula so that once it crosses the MV it can be angled downward in the direction of the mitral plane. Again, TEE imaging is of utmost importance to guide the operator during LV navigation and valve crossing. In our experience, we found it very useful to direct the AngioVac cannula using the bi-plane view.

Another drawback of this procedure is the risk of stroke due to mass embolization. For this reason, we decided to position a cerebral embolism protection device as reported by other authors (5, 6). This procedure is easy and safe and does not significantly prolong the fluoroscopy time of the procedure. Femoral or radial access can be used depending on the operator's preference and reinfusion cannula position.

Contrary to previous reports, we preferred an arterial reinfusion site (5), and we did not establish a parallel ECMO circuit (4) to support hemodynamics since the patient was not in septic shock and presented good cardiovascular conditions; however, we included an oxygenator that increased the filter efficacy of the system. For this reason, an ACT > 450 s was achieved with no significant periprocedural bleeding complications. Other groups (5) preferred complete venous access with transeptal aspiration and reinfusion in the femoral vein. Even if this option was safe in reducing the risk of vascular complications, we believe that such a setup might provide inferior hemodynamic support to the patient, overloading the right system. However, in the case of inadequate arterial access for reinfusion, this choice would be preferable.

## Conclusions

A minimally invasive approach using the AngioVac system can be safe and effective to treat native mitral valve and aortic and mitral prosthesis endocarditis in selected patients, especially when prohibitive surgical risk is present. The combined use of cerebral embolic protection is of utmost importance.

## Data Availability

The raw data supporting the conclusions of this article will be made available by the authors, without undue reservation.
